# Supplementary material for: Differentiating Functional Cognitive Disorder from Early Neurodegeneration: A Clinic-Based Study
Source: Brain Sci. 2021 Jun 17;11(6):800. doi: 10.3390/brainsci11060800 (PMC8234331; doi:10.3390/brainsci11060800)
Supplement: Supplementary file 1 [file brainsci-11-00800-s001.zip › brainsci-1247320-supplementary.pdf]

Supplementary material. Table S1. MMPI-2-RF profiles (FCD group only)

Using the full sample of 20 participants with FCD, the mean number of items unscorable (including marked “cannot say”) was 19 (standard error 7.8), out of a total of 338 items. The VRIN score (amongst those with at least 90% response rate for VRIN items) was 58.6 (standard error 3.4). The TRIN score (amongst those with at least 90% response rate for TRIN items) was 60.3 (standard error 2.1).

For the results in the table below, participants were only included if there was no evidence of possible variable or fixed reporting, and if they gave at least 90% valid responses for the scale in question.

**Scale**	**T-score**	**Standard Error**	**n**
Validity			
FR	75.4	5.9	18
FPR	58.9	4.5	18
FS	73.6	5.1	18
FBSR	66.9	4.1	18
LR	60.8	2.5	17
KR	45.6	1.6	18
Higher-order			
EID - Emotional/Internalizing Dysfunction	59.6	3.3	17
THD - Thought Dysfunction	57.8	2.6	16
BXD - Behavioral/Externalizing Dysfunction	46.0	2.4	18
RC (Restructured Clinical)			
RCd – Demoralization	60.4	2.8	18
RC1 - Somatic Complaints	70.6	4.5	17
RC2 - Low Positive Emotions	61.8	4.7	16
RC3 – Cynicism	52.1	1.7	17
RC4 - Antisocial Behavior	47.4	2.1	18
RC6 - Ideas of Persecution	55.7	2.6	17
RC7 - Dysfunctional Negative Emotions	55.0	2.6	17
RC8 - Aberrant Experiences	63.8	2.6	16
RC9 - Hypomanic Activation	47.8	2.6	17
Somatic/cognitive			
MLS – Malaise	66.3	3.4	18
GIC - Gastrointestinal Complaints	60.2	4.2	18
HPC - Head Pain Complaints	63.6	3.0	18
NUC - Neurological Complaints	71.4	4.6	17
COG - Cognitive Complaints	76.7	3.7	16
Internalizing Scales			
SUI - Suicidal/Death Ideation	59.7	5.4	15
HLP - Helplessness/Hopelessness	58.5	3.0	17
SFD - Self-Doubt	58.6	2.6	17
NFC – Inefficacy	56.2	2.5	16
STW - Stress/Worry	52.8	3.4	15
AXY – Anxiety	64.6	5.3	16
ANP - Anger Proneness	51.8	1.9	17
BRF - Behavior-Restricting Fears	61.5	3.8	18
MSF - Multiple Specific Fears	47.6	1.3	16
Externalising			
JCP - Juvenile Conduct Problems	48.3	2.1	17
SUB - Substance Abuse	45.9	1.6	17
AGG – Aggression	47.7	2.4	17

ACT – Activation	48.6	1.9	17
Interpersonal Scales			
FML - Family Problems	52.4	3.0	17
IPP - Interpersonal Passivity	50.6	2.2	15
SAV - Social Avoidance	58.3	3.6	16
SHY – Shyness	50.3	2.2	14
DSF – Disaffiliativeness	59.3	3.3	16
Interest Scales			
AES - Aesthetic-Literary Interests	42.9	2.6	16
MEC - Mechanical-Physical Interests	46.3	2.3	16
Personality Psychopathology Five (PSY-5) Scales			
AGGR-r - Aggressiveness-Revised	49.6	2.3	16
PSYC-r - Psychoticism-Revised	58.1	2.0	17
DISC-r - Disconstraint-Revised	44.2	2.4	17
NEGE-r - Negative Emotionality/Neuroticism-Revised	56.4	3.7	18
INTR-r - Introversion/Low Positive Emotionality-Revised	61.2	4.4	17
